# Involvement of P-gp on Reversing Multidrug Resistance Effects of 23-Hydroxybetulinic Acid on Chemotherapeutic Agents

**DOI:** 10.3389/fphar.2021.796745

**Published:** 2021-12-15

**Authors:** Zhihao Liu, Xiaozhou Wen, Guangji Wang, Ying Zhou

**Affiliations:** ^1^ Department of Pharmacy, The First Affiliated Hospital of Nanchang University, Nanchang, China; ^2^ Department of Clinical Pharmacology, College of Pharmacy, Dalian Medical University, Dalian, China; ^3^ Key Laboratory of Drug Metabolism and Pharmacokinetics, China Pharmaceutical University, Nanjing, China; ^4^ Jiangsu Province Hospital of Chinese Medicine, Nanjing, China; ^5^ Affiliated Hospital of Nanjing University of Chinese Medicine, Nanjing, China

**Keywords:** betulinic acid, multidrug resistance, P-gp, 23-hydroxybetulinic acid, cytotoxicity

## Abstract

Betulinic acid (BA) and 23-Hydroxybetulinic acid (23-HBA) are natural products with similar structures, which show a range of biological effects including cytotoxicity activity. The aim of current research was to investigate and evaluate the combinational cytotoxicity of BA and 23-HBA with chemotherapeutic agents *in vitro*, and to clarify the potential interaction and related mechanism with P-gp. Instead of BA, 23-HBA could increase cytotoxicity of MCF-7/ADR cells to adriamaycin (ADR) and vincristine (VCR). The intracellular accumulation of ADR/VCR in MCF-7/ADR cells was obviously increased in the presence of 23-HBA. Furthermore, 23-HBA could show dose-dependent increase on the transport of VCR and digoxin, which are typical P-gp substrates, in both MDCK-MDR1 and Caco-2 cells. However, the transport of BA and 23-HBA was not influenced by P-gp inhibition in MDCK-MDR1 cells. MDR1 shift assay and molecular docking model suggested that both compounds showed interaction with P-gp, yet the binding affinity and sites are different. In conclusion, 23-HBA could strongly improve the efficacy of anti-tumor agents in multidrug resistance (MDR) cells, which was related to P-gp inhibition. The MDR1 shift assay and molecular docking study further revealed that 23-HBA and BA showed different interaction modes with P-gp.

## Introduction

The incident of malignant tumors is a frequent disease which widely threatens human health, and the death rate of tumors is second in all diseases, exceeded only by heart disease (Masoudkabir et al., 2017). Until now, special effective treatments for tumors haven’t be found, and chemotherapy is still an effective and widely used approach for curing malignant tumors (Zarros et al., 2018). However, during the long-term applications, chemotherapeutics include side effects and the emergence of drug resistance (Maino et al., 2000; Alfarouk et al., 2015). It is an urgent to search for novel antitumor drugs or ancillary drugs for pharmaceutical researchers.

Multidrug resistance (MDR) could lead to low efficiency of chemotherapeutic agents that are mechanistically and/or structurally unrelated and therefore strongly cause treatment effects of drugs (Xue et al., 2016; Sun et al., 2019). The mechanisms of MDR are various, yet, the most acceptable reason would be the high expression of ATP-binding cassette (ABC) transporters (Nanayakkara et al., 2018). P-glycoprotein (P-gp) is one of the most investigated ATP-dependent transmembrane transporters (Li et al., 2014). Besides its role in cancer cell resistance, P-gp has multiple physiological functions as well, since it’s expressed also in many important non-tumoural tissues (Zhang et al., 2018). It was suggested that in tumors, P-gp are expressed not only in cell membranes but also in membranes of some subcellular organelles (such as mitochondria and nucleolus) (Munteanu et al., 2006). These organelles are target points of most antineoplastic. P-gp, which is overexpressed in tumor cells, could increase the excretion, reduce the concentration of chemotherapeutic agents in target cells and organelles, and finally generate drug resistance (Wang et al., 2016). Considering the key role of P-gp in MDR, developing P-gp inhibitors is of great clinical significance, since it could reduce dosage of antineoplastic and raise curative effect (Jia et al., 2016). Natural products and their derivatives with P-gp inhibitory property have been paid more and more attention in recent years, such as glycyrrhitic acid, emodin, and ginsenoside (Li et al., 2014; Alfarouk et al., 2015), which show potency and multitarget compared with traditional P-gp inhibitors (Dewanjee et al., 2017).

Betulinic acid (BA) was first isolated from the East African evergreen tree, Ziziphus mauritiana (Fong et al., 1995). It is a naturally occurring pentacyclic triterpenoid, which has revealed cytotoxicity effects against some specific tumors (like melanoma, head and neck squamous cell carcinoma, etc.) (Fulda, 2008; Saeed et al., 2018). Previous reports suggested that, besides inducing apoptosis, it could also act as a chemosensitizer and radiosensitizer in some tumors (Fulda and Kroemer, 2009). The mechanism is not clear; furthermore, whether it’s relevant to some transporters (such as P-gp) hasn’t been reported yet. One type of opentacyclic triterpenes, 23-hydroxybetulinic acid (23-HBA), is an analogue of BA (Zhou et al., 2019). It’s the important active component of Pulsatilla chinensis (Bunge) Regel, which shows “blood-cooling” and detoxification activities. And, 23-HBA has been reported to express cytotoxicity against various tumors and HIV and could restrain vascularization. In the previous study, it was confirmed that 23-HBA could enhance the cytotoxicity of cancer cells to current antitumor agents, which was associated with regulating P-gp expression and function (Zheng et al., 2010).

However, still unknown is the mechanism of P-gp on reversing MDR effects by 23-HBA on chemotherapeutic agents and whether 23-HBA and BA perform similar mechanism of reversing MDR. This will be the goal concerned in the present research. Firstly, we studied the inversion effect of MDR by noncytotoxic BA and 23-HBA in MCF-7 and MCF-7/ADR cells; then, we compared P-gp inhibition activities of 23-HBA and BA by using Caco-2 cells and MDCK-MDR1 cells, which are both “golden models” to investigate P-gp mediated drug disposition (Sun and Pang, 2008; Liu and Liu, 2016); in the last, we studied the structure-activity relationship of BA and 23-HBA with P-gp by MDR1 shift assay, molecular docking, and MDCK-MDR1 monolayers.

## Materials and Methods

### Chemicals

Betulinic acid (BA, purity ≥98%) was obtained from Shanghai Ronghe Medicine Science and Technology Co., Ltd. (China). In addition, 23-hydroxybetulinic acid (23-HBA, purity ≥99.8%) was obtained from Professor Wencai Ye at Jinan University. Verapamil hydrochloride was obtained from the National Institute for the Control of Pharmaceutical and Biological Products (China). Adriamycin (ADR) was obtained from Zhejiang Hisun Pharmaceutical Co., Ltd. (China). Vincristine (VCR) was purchased from Hangzhou Minsheng Pharmaceutical Group Co., Ltd. (China). All other solvents and reagents were of analytical grade.

### Cell Cultures

MCF-7 (human breast carcinoma cells) and MCF-7/ADR (P-gp-overexpressing derivative cells) cell lines were obtained from Blood Diseases Hospital, Chinese Academy of Medica (Shi et al., 2011). The cells were routinely maintained in RPMI 1640 with 10% heat-inactivated fetal bovine serum (FBS), 1% non-essential amino acid solution, 0.1 mg/ml streptomycin, and 100 units/ml penicillin. Caco-2 cells were purchased from American Type Culture Collection (ATCC, Rockville, MD, United States). Vector-MDCK and MDR1-MDCK cells were purchased from PharmaResources Co., Ltd. (China). Both Caco-2 and MDCK cells were maintained at Dulbecco’s modified Eagle’s medium (DMEM) with 10% FBS, 1% non-essential amino acid solution, 0.1 mg/ml streptomycin, and 100 units/ml penicillin. MCF-7/ADR cells were treated with 2 mg/ml ADR (2 mg/ml) and were cultured in ADR-free medium for more than 14 days before the experiment. All cells were cultured at 37°C in a humidified atmosphere of 5% CO_2_.

### Trypan Blue Exclusion Assay

Trypan blue exclusion assay was used to measure the viability of cells to compounds. Briefly, MCF-7/sensitive (MCF-7/S) and MCF-7/ADR cells were diluted into the density of 0.5×10^4^ cells/ml, then cells (1 ml) were seeded in culture plates. After 24 h, cells were given ADR (0.08 µM for MCF/S, 3 µM for MCF/ADR) or VCR (0.5 µM for MCF/S, 6 µM for MCF/ADR) alone, or in combination with series concentrations of BA or 23-HBA (0.2, 2 and 20 µM), and were cultured at 37°C for 48 h. The cells were exposed to trypan blue assay (Gibco, Carlsbad, CA, United States) in a ratio of 1:9. The viable and dead cells (stained in blue) were counted by haemacytometer.

### Hoechst Assay

MCF-7/ADR cells were collected and cultured at the density of 2.0 × 10^5^/well in 24-well plates. Then the cells were exposed to ADR (3 μM) without or with BA/23-HBA (0.2, 2, and 20 µM). The cells were dyed with Hoechst 33,342 (Beyotime, Shanghai, China) in the dark at 37°C for 30 min after washing with phosphate buffered saline (PBS). The morphological changes of the nucleus were evaluated by a fluorescence microscope.

### Adriamycin/Vincristine Accumulation Study

MCF-7 cells (1×10^5^/Well) were incubated in 24-well plates. Then, for both ADR and VCR groups, the cells were given ADR/VCR (5 µM) without or with verapamil (20 µM) or BA/23-HBA (0.2, 2, and 20 µM). After 1 h incubation, the compound solution was aspirated to stop further accumulation, and the monolayers were rinsed with cold Hank’s solution for three times. The drug content was detected by lysing the cells with ultrasound, and then the single well lysate was transferred to a plastic EP tube for further analysis. The protein concentration of the cells was measured using Coomassie brilliant blue.

### Caco-2 Uptake Study

The Caco-2 cells uptake study was taken as described previously with minor modifications (Liu et al., 2011). Briefly, the cells (1×10^5^/Well) were cultured in 24-well plates, and culture medium was changed every other day. The uptake study was taken when the monolayers formed. For digoxin groups, the cells were given digoxin (5 µM) with or without verapamil (20 µM) or BA/23-HBA (0.2, 2, and 20 µM). For vincristine groups, the cells were given VCR (2 µM) with or without verapamil (20 µM) or BA/23-HBA (0.2, 2, and 20 µM). After 2 h incubation, the drug solution was removed to terminate reaction, and the monolayers were washed three times with cold Hank’s solution. The other steps were taken according to the above process in *Adriamycin/Vincristine Accumulation Study*.

### MDCK-MDR1 Transport Study

The MDCK-MDR1 cells transport study was taken as described previously with minor modifications (Liu et al., 2016). Briefly, MDCK-MDR1 cells (1×10^5^/Well) were seeded onto 0.4 mm pore-size transwell inserts (Millipore, MA, United States) in 24-well plates. Cells were cultured for 5–7 days, when the transepithelial electrical resistance (TEER) values were 120–240 Ω cm^2^.

Firstly, transport study was taken to determine the inhibition effects of the BA/23-HBA on P-gp. The cells were rinsed with warmed Hank’s balanced salt solution (HBSS), and the inserts were incubated for 20 min at 37°C. Then the transport was started by adding HBSS containing known P-gp substrate digoxin (5 µM) with or without BA/23-HBA (0.2–20 µM) to either apical or basolateral side of monolayer. Aliquots (50 µl) were taken from the receiver compartment at intervals of 30, 60, 90, and 120 min and replaced with fresh buffer. In the study, verapamil (VER), known P-gp inhibitor, was selected as positive control. Secondly, the transport study was taken to certify whether BA/23-HBA are substrates of P-gp. The study was initiated by adding HBSS containing BA/23-HBA (0.2–20 µM) with or without verapamil (20 µM) to either apical (A) or basolateral (B) side of monolayer. The other steps were taken according to the above process.

### MDR1 Shift Study

The MDR1 shift assay was taken according to the manufacturer’s protocol (Millipore Bioscience Research Reagents, Temecula, CA) and previous report (Zhang et al., 2010). Briefly, MCF-7/ADR cells (1 × 10^6^ cells/ml) were collected and exposed in UIC2 binding buffer followed by pre-incubated at 37°C for 10 min, then reaction was started by adding BA/23-HBA (25 μM) at 37°C for another 10 min. The monoclonal antibody UIC2 (2.5 μl) was added and incubated for 15 min, then 1 ml of cold binding buffer was used to terminate reaction. The cells were rinsed twice, followed by adding cold phycoerythrin-labeled anti-mouse IgG (250 μl) for another 15 min in the dark. Finally, cells were rinsed and incubated in cold binding buffer (250 μl) and further evaluated by flow cytometer (Cytomics FC 500; Beckman Coulter, Fullerton, CA). In this study, vincristine (25 µM) was set as positive control.

### Molecular Docking Study

The binding mechanisms of BA/23-HBA with P-gp were evaluated by molecular docking approach with AutoDock 4.2 in Sybyl version. The crystal structure of MDR1A/P-gp (PDB code: 3G60) was selected in the docking approach (Liu et al., 2016). In the processing, all the ligand residues were permitted to shift while the structure of P-gp protein was fixed. The conformations with lowest binding energy were chosen for further mechanism evaluation.

### Analysis Method

See supporting information Supplementary Material S1.

### Data Analysis

The apparent permeability values (P_app_) were evaluated in all experiments according to the following equation:

$$P_{app}=\frac{dQ/dt}{AC_{0}}$$ (1)

where A means the insert area and C_0_ represents the initial concentration. dQ/dt represents the slope of the cumulative content transported during the time course of the period studied.

The efflux ratio (ER) was detected according to the ratio of P_app_ as the following equation:

$$\mathrm{ER}=\frac{P_{app}\left(B-A\right)}{P_{app}\left(A-B\right)}$$ (2)

The individual point was expressed by mean ± S. D unless indicated otherwise. The statistical analysis between different groups was performed by non-paired *t*-test. Values with *p* < 0.05 were considered as statistically significant.

## Results

### Effects of BA/23-HBA on Potentiating Cytotoxicity of MCF-7 Cells to ADR or VCR

**FIGURE 1:**
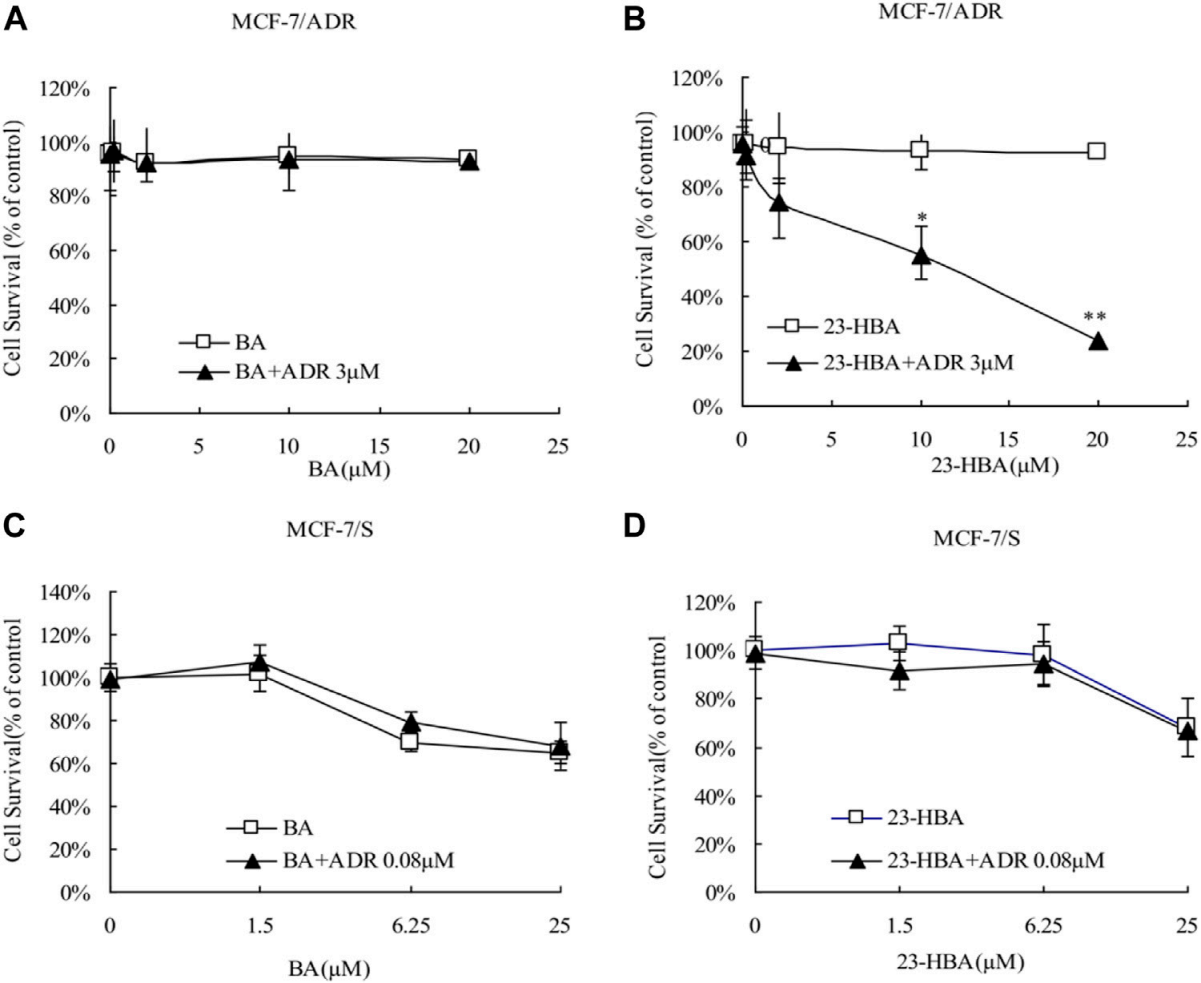
Effects of 23-HBA and BA on the cell survival of MCF-7/ADR (**A** and **B**) and MCF-7/S (**C** and **D**) cells to ADR. The cells were incubated for 48 h with ADR in the absence or presence of three different concentrations of 23-HBA (or BA), and cell growth inhibition was determined by Trypan Blue counting assays. *p < 0.05, **p < 0.01, ***p < 0.001 vs. control.

**FIGURE 2:**
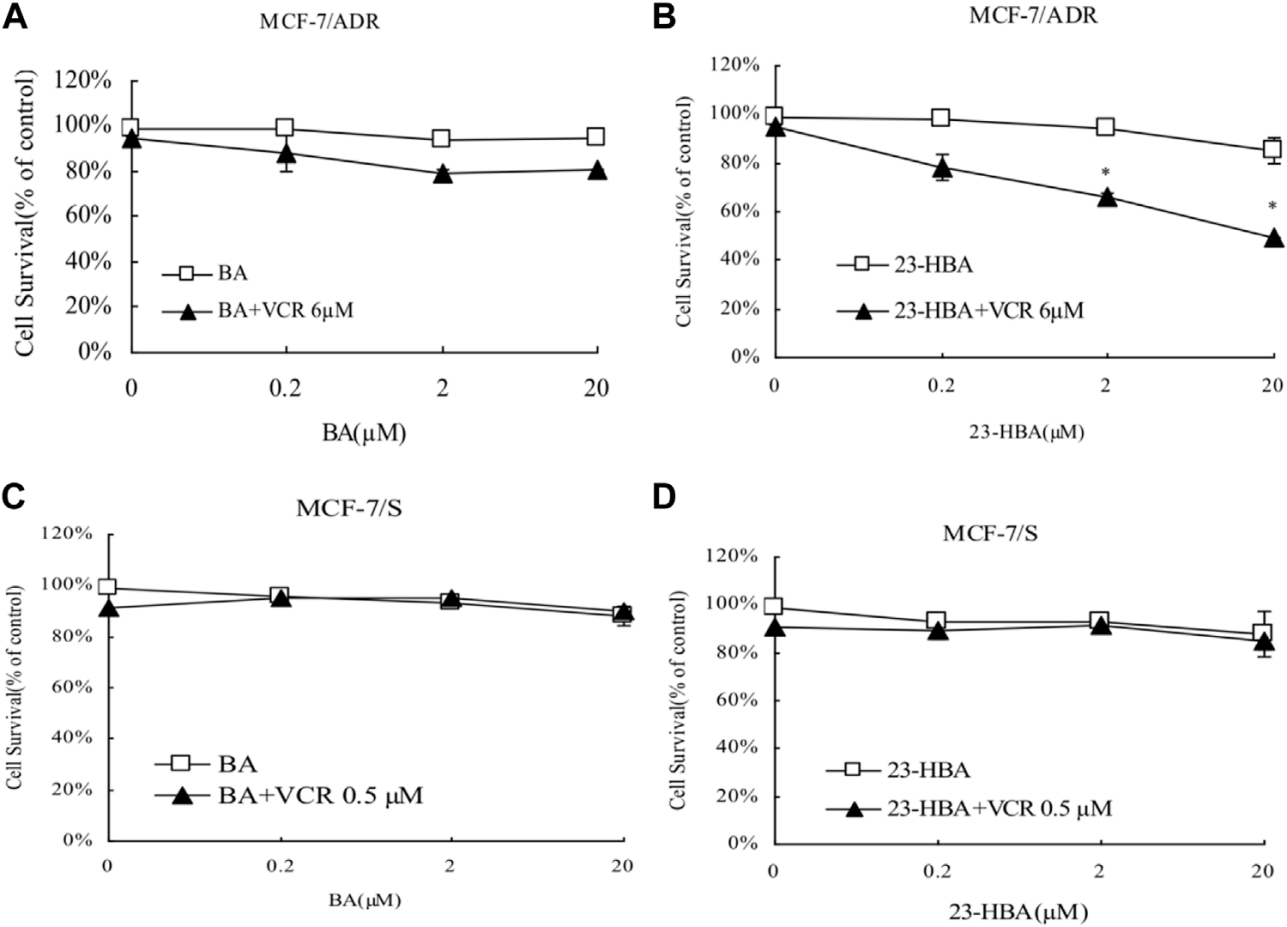
Effects of 23-HBA and BA on the cell survival of MCF-7/ADR (**A** and **B**) and MCF-7/S (**C** and **D**) to VCR. Cells were treated for 48 h with VCR and three different concentrations of 23-HBA (or BA), and cell growth inhibition was detected by Trypan Blue counting assays. *p < 0.05, **p < 0.01, ***p < 0.001 vs. control.

Instead of BA, 23-HBA can increase cytotoxicity of MCF-7/ADR cells to ADR or VCR in concentration-dependent manner (Figures 1A,B, Figures 2A,B). However, for MCF-7/S cells, neither 23-HBA nor BA showed significant effects on cytotoxicity in MCF-7/S cells (Figures 1C,D, Figures 2C,D).

**FIGURE 3:**
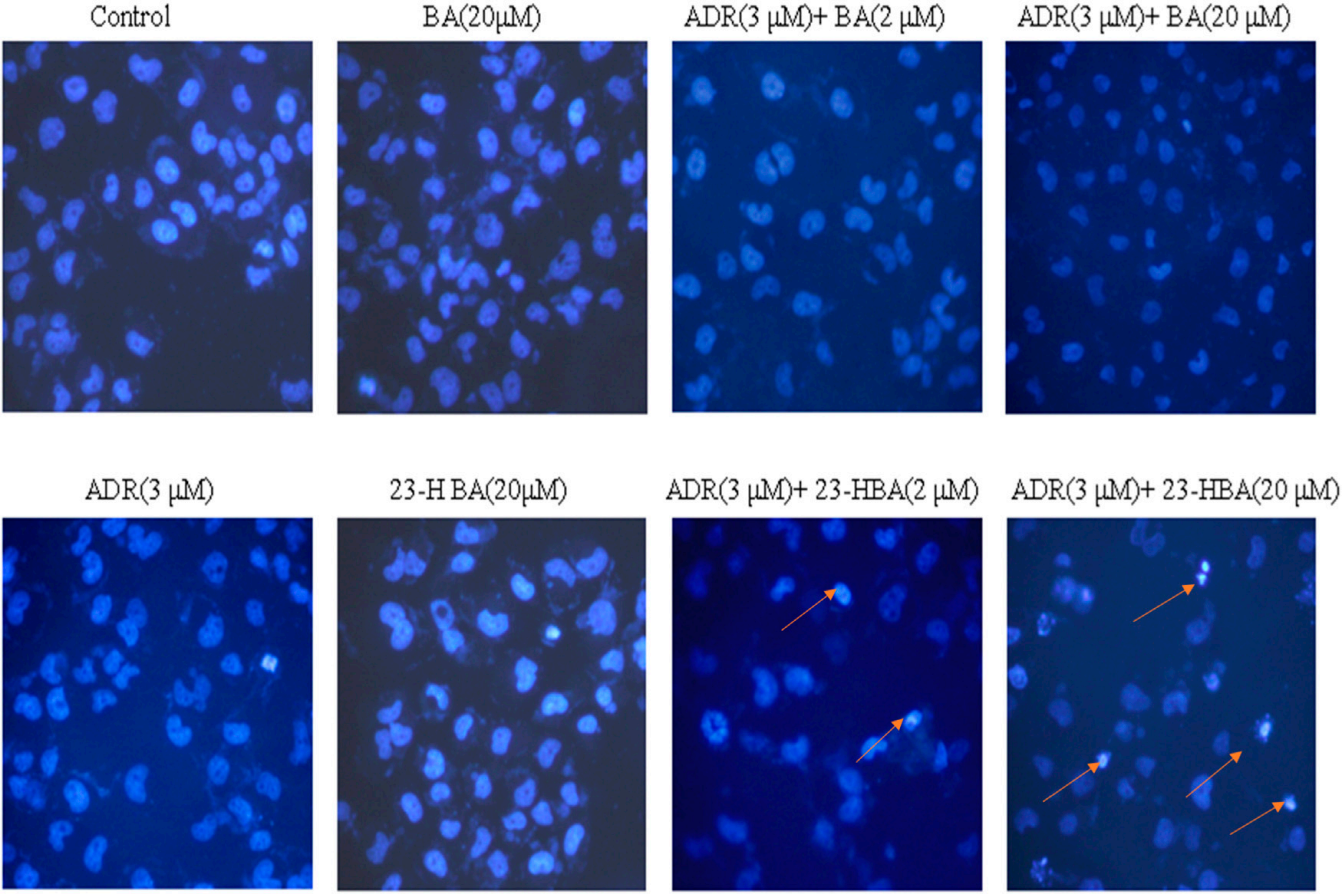
Fluorescence intensity of ADR (3 µM) in the presence or absence of different concentrations of 23-HBA and BA (2 and 20 µM) in MCF-7/ADR cell. Cells were treated with 3 µM of ADR and 23-HBA (2 and 20 µM) for 2 h. Then cells were fixed with 4% formaldehyde. After staining with Hoechst 33,342, the fluorescence intensity of ADR was determined by high content analysis (200×). Arrowheads indicate the apoptosis cells.

In addition, Hoechst assay was further used to detect effects of the combination exposure on the apoptosis of MCF-7/ADR cell line. The number of apoptosis cells in BA (20 μM), 23-HBA (20 μM), or ADR (3 μM) group was not obviously different with the control group (Figure 3). However, in 23-HBA combination exposure group, 23-HBA could produce a dose-dependent (2 and 20 μM) increase in the apoptosis caused by ADR (3 μM). All results suggested that the combination exposure of 23-HBA revealed a stronger effect on increasing the cytotoxicity of resistant cells to ADR or VCR.

### Effects of BA/23-HBA on the Uptake of ADR/VCR in MCF-7 Cells

**FIGURE 4:**
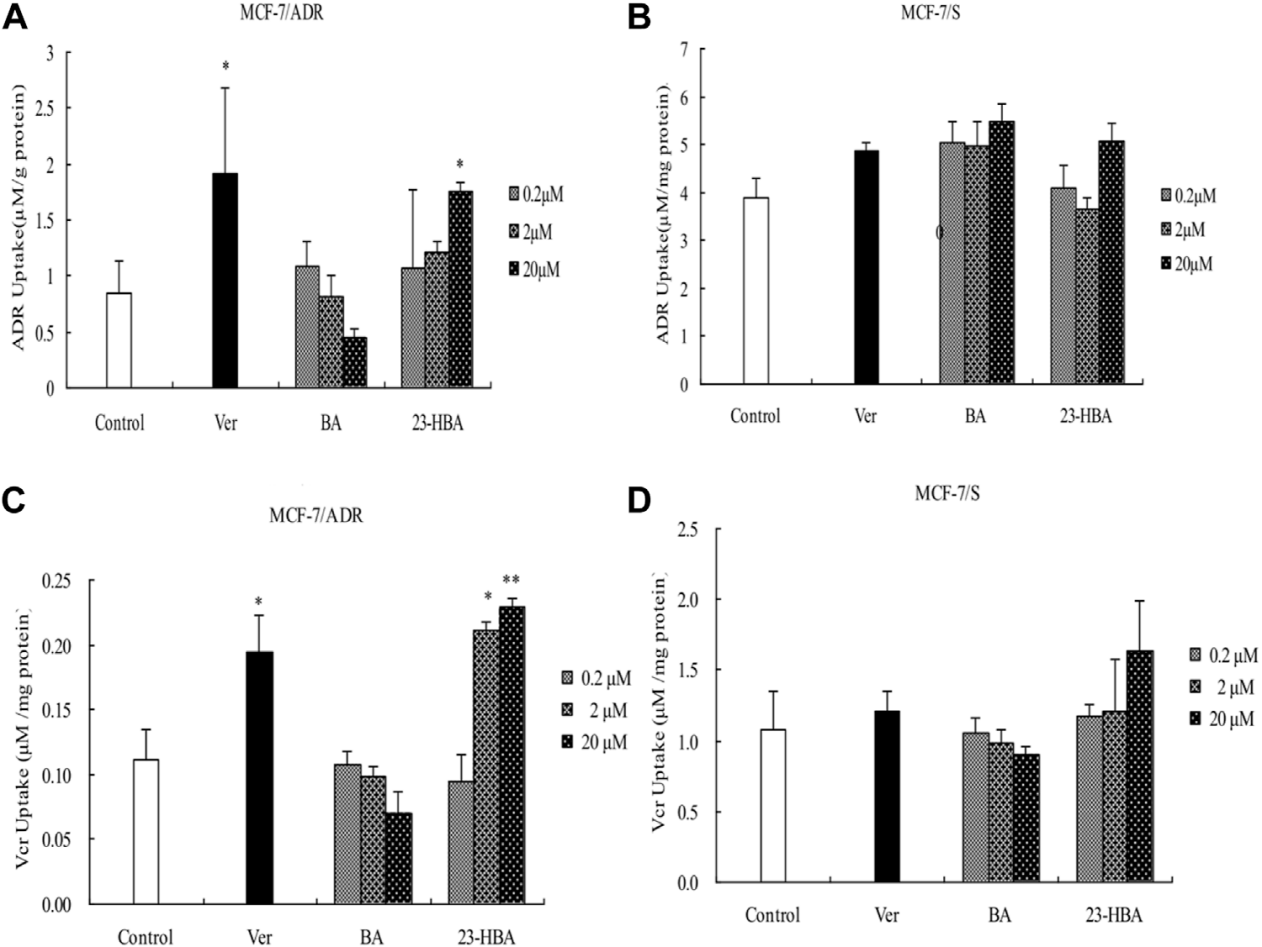
Effects of 23-HBA and BA on the intracellular accumulation of ADR (**A** and **B**) and VCR (**C** and **D**) in MCF-7/ADR and MCF-7/S cells, respectively. Intracellular accumulation of ADR/VCR(5 µM) with three different concentration of 23-HBA or BA. Verapamil (20 µM) was used as positive control. *p < 0.05, **p < 0.01, ***p < 0.001 vs. control.

To understand the mechanism and cytotoxicity difference between BA and 23-HBA, the accumulation of ADR/VCR with or without BA/23-HBA was measured in MCF-7 cells. The results confirmed that accumulation amouts of ADR/VCR in MCF-7/S cells were greater than that of MCF-7/ADR cells (Figures 4A,C). In addition, instead of BA, intracellular accumulation of ADR/VCR was obviously increased in the presence of 23-HBA in resistant MCF-7 cells, which was similar with typical P-gp inhibitor verapamil (Figures 4A,C). However, little impact was observed on the accumulation of ADR/VCR in the sensitive MCF-7 cells with or without BA/23-HBA (Figures 4B,D). In summary, these results suggested that 23-HBA would inhibit P-gp-involved accumulation in resistant MCF-7 cells.

### Effects of BA/23-HBA on the Activity of P-gp in Caco-2 Cells

**FIGURE 5:**
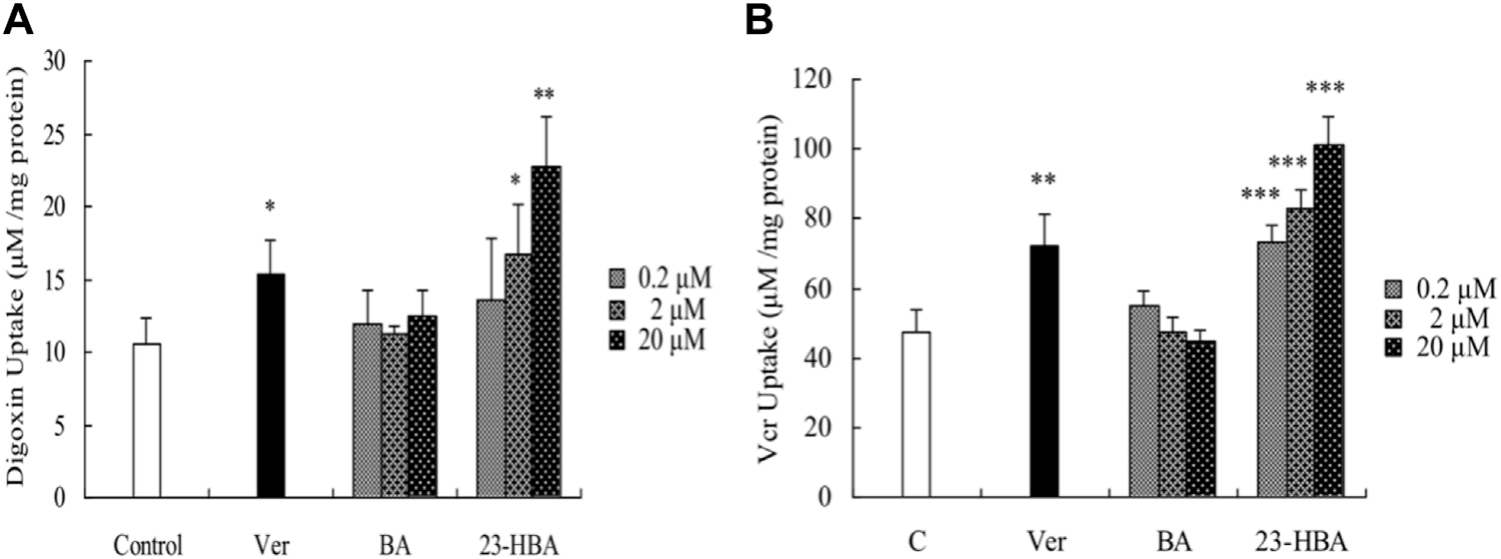
Effects of 23-HBA and BA on the intracellular accumulation of digoxin **(A)** and VCR **(B)** in Caco-2 cells. Intracellular accumulations of digoxin or VCR (5 µM) in the absence or presence of 23-HBA or BA (0.2, 2, 20 µM) in Caco-2 cells. Verapamil (20 µM) was used as positive control. *p < 0.05, **p < 0.01, ***p < 0.001 vs. control.

To elucidate the interaction of BA/23-HBA on the function of P-gp, the uptake of VCR was measured in Caco-2 cells. Also, 23-HBA (0.2, 2, and 20 μM) showed dose-dependent increase on the penetration of VCR and digoxin (Figure 5), which were known substrates of P-pg. In addition, the inhibitory effect of 23-HBA (20 μM) was higher than that of verapamil (20 μM), which was a typical inhibitor of P-gp. However, almost no obvious inhibition was observed for BA at different concentrations. The results suggested that 23-HBA, instead of BA, could be a potential inhibitor of P-gp.

### Interaction Between BA/23-HBA and P-gp Across MDCK-MDR1 Cell Monolayers

To further confirm the interaction between BA/23-HBA and P-gp, the transcellular transport study was performed in MDCK-MDR1 cell model. The transports of digoxin from basal (B) to apical (A) were greater than those from apical (A) to basal (B) for all three concentrations used in MDCK-MDR1 cells, and this direction could be partly reversed when 23-HBA was in combination (Table 1). The results suggested that the model is successful and further confirmed that 23-HBA, instead of BA, was a potential inhibitor of P-gp. As expected, 23-HBA could dose-dependent increase the penetration (A-B) and decrease efflux (B-A) of VCR, and thus reversed the transport direction represented by efflux ratio (ER) values (Table 2). The results suggested that the 23-HBA would inhibit the activity of P-gp and then influence the polar transport of VCR. In addition, further study was taken to verify whether BA and 23-HBA were substrates of P-gp in MDCK-MDR1 cells. The ER values for both BA and 23-HBA were less than 1.5 at all the concentrations; however, verapamil could partly influence the penetration and efflux (Table 3). The results suggested that P-gp could slightly influence the transport of BA and 23-HBA, yet the impact was limited.

**TABLE 1:** Apparent permeability coefficient (Papp) and net efflux (R_E_) of bi-directional digoxin transport across polarized MDCK-MDR1 cell monolayers.

	Papp (×10^−6^ cm s^−1^)	
	A→B	B→A	R_E_	Folds (R_E_)
Control	0.31 ± 0.05	11.32 ± 0.45	36.56	
VER (µM)				
20	1.32 ± 0.14***	9.42 ± 0.50***	7.14	5.12
23-HBA (µM)				
0.2	0.40 ± 0.07	12.56 ± 0.96	31.57	1.16
2	0.48 ± 0.05*	13.43 ± 2.27	27.96	1.31
20	1.81 ± 0.21***	4.86 ± 0.59***	2.69	13.61
BA (µM)				
0.2	0.37 ± 0.09	10.89 ± 0.42	29.11	1.26
2	0.64 ± 0.06	10.91 ± 0.64	17.06	2.14
20	0.55 ± 0.18	12.92 ± 2.61	23.42	1.56

Data are presented as mean ± SEM, *n* = 3. A→B, apical to basal; B→A, basal to apical. Net efflux was calculated as the ratio of P_app_ from B→A to A→B. *p < 0.05, **p < 0.01, ***p < 0.001 vs. control.

**TABLE 2:** Apparent permeability coefficient (Papp) and net efflux (R_E_) of bi-directional VCR transport across polarized MDCK-MDR1 cell monolayers.

	Papp (×10^−6^ cm s^−1^)	
	A→B	B→A	R_E_	Folds (R_E_)
Control	0.35 ± 0.17	3.86 ± 0.71	10.89	
VER (µM)				
20	0.75 ± 0.03^*^	3.49 ± 0.39	4.68	2.33
23-HBA (µM)				
0.2	0.69 ± 0.30	4.45 ± 1.12	6.41	1.70
2	2.49 ± 0.59^**^	0.39 ± 0.01^**^	0.16	70.15
20	2.19 ± 1.74^**^	0.48 ± 0.08^*^	0.22	49.32
BA (µM)				
0.2	0.45 ± 0.06	3.73 ± 0.07	8.30	1.31
2	0.42 ± 0.15	3.88 ± 1.04	9.18	1.19
20	0.42 ± 0.13	3.83 ± 1.01	9.11	1.20

Data are presented as mean ± SEM, *n* = 3. *p < 0.05, **p < 0.01, ***p < 0.001 vs. control.

**TABLE 3:** Apparent permeability coefficient (Papp) and net efflux (R_E_) of bi-directional transport of 23-HBA and BA with or without verapamil across polarized MDCK-MDR1 cell monolayers.

	Papp (×10^−6^ cm s^−1^)	
	A-B	B-A	R_E_	Folds (RE)
23-HBA(µM)				
0.2	6.73 ± 0.09	10.01 ± 1.38	1.49	
2	7.07 ± 0.64	8.75 ± 0.31	1.24	
20	5.95 ± 0.94	4,28 ± 0.72	0.72	
23-HBA(µM) +VER				
0.2	8.49 ± 1.39	5.20 ± 0.59	0.61**	0.41
2	10.03 ± 3.22	7.83 ± 0.32	0.78*	0.63
20	12.76 ± 1.13	7.47 ± 1.64	0.59	0.82
BA (µM)				
0.2	61.23 ± 9.36	55.09 ± 9.80	0.90	
2	5.43 ± 0.50	6.51 ± 0.81	1.20	
20	0.72 ± 0.26	0.59 ± 0.14	0.82	
BA (µM) +VER				
0.2	104.54 ± 10.47	62.13 ± 4.71	0.59*	0.66
2	7.65 ± 1.53	6.90 ± 0.76	0.90	0.75
20	0.26 ± 0.05	0.17 ± 0.05	0.64	0.78

Data are presented as mean ± SEM, *n* = 3. *p < 0.05, **p < 0.01, ***p < 0.001 vs. control.

### Inhibitory Mechanisms of BA/23-HBA on P-gp

**FIGURE 6:**
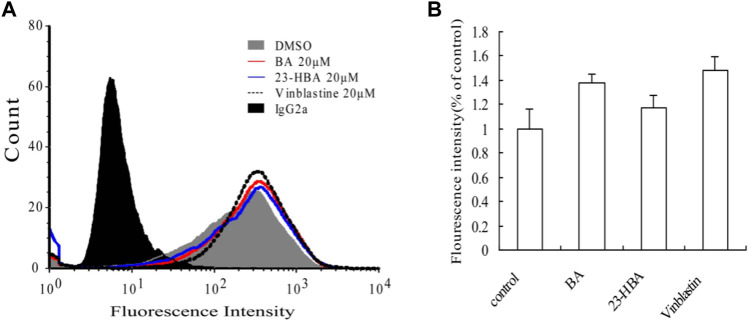
Effects of BA and 23-HBA on the binding of conformation-sensitive antibody UIC2 to P-gp. **(A)**, effects of compounds on the binding conditions. The cells treated with normal IgG2a were used as a background control, and DMSO treatment was selected as a solvent control. **(B)**, results quantified from three independent experiments are presented as mean ± SEM.

MDR1 shift assay was performed to confirm whether BA/23HBA could alter the P-gp conformation. The P-gp conformation-specific antibody, UIC2, was selected to predict reasonable structure change by P-gp substrates. The waves with compound treatment were shifted as compared to solvent control (Figure 6A). Additionally, fluorescence intensity showed slight increase between compound treatment and solvent which was in line with the positive control vinblastine (Figure 6B), suggesting that BA/23-HBA would be a substrate of P-gp with low binding affinity.

### Molecular Docking Mechanism Between BA/23-HBA and P-gp

**FIGURE 7:**
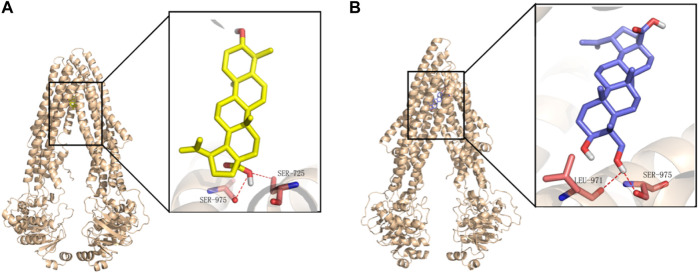
Binding mode of BA **(A)** and 23-HBA **(B)** in the active site of P-gp. Yellow for BA, blue for 23-HBA, and magenta for binding residues of P-gp.

To further clarify the interaction mechanism between P-gp and BA/23-HBA in molecular perspective, docking simulation was performed as the method described above. BA (−9.04 kcal⋅mol^−1^) docked with slightly higher binding energy to the structure pocket, compared to 23-HBA (−8.07 kcal⋅mol^−1^). Both BA and 23-HBA could form hydrogen bonds with residues of P-gp (Figure 7). Particularly, BA could bind with SER975 and SER725 through the carboxyl on the site 28. However, 23-HBA bond with LEU 971 and SER 975 through hydroxy on the site 23. It is interesting that the binding orientation of the compounds with residues is different between BA and 23-HBA; however, the cause of this phenomenon is unknown. The results suggested that both BA and 23-HBA could show affinity with P-gp; however, the binding sites and scales were different between them.

## Discussion

Until now, selection of alternative drugs from natural constituents would be a reasonable choice to decrease drug resistance via P-gp and to promote cytotoxicity to cancer cells (Zhang et al., 2012; Sun et al., 2019). To investigate the role of BA/23-HBA to overcome MDR, its ability to re-sensitize MCF-7/ADR cells was evaluated. Current study suggested that no cell toxicity was detected when BA or 23-HBA was used at 0–20 μM as a single agent. However, combination exposure of 23-HBA (0–20 μM) and ADR (3 μM) showed significant cytotoxicity to MCF-7/ADR (Figure 1B), which suggested that 23-HBA could dose-dependent reduce the resistance of MCF-7/ADR cells to ADR. Inconsistently, this synergistic effect mentioned above was not observed for BA (0–20 μM) and ADR (3 μM) (Figure 1A). As reported, MCF-7/ADR cells were confirmed to be ADR-resistant with the mechanism such as overexpression of protein and mRNA levels of P-gp (Chen et al., 2015). Thus, 23-HBA could be a P-gp-included MDR reversal natural product, and this point was further verified by 23-HBA-induced reduction to VCR resistance in resistant MCF-7/ADR cells (Figure 2). As expected, 23-HBA, instead of BA, could enhance the ADR induced apoptosis on MCF-7/ADR cells in a concentration-dependent style (2 and 20 μM) in Hoechst assay (Figure 3). However, the same effects did not appear when given 23-HBA or ADR as single agent. This further confirmed that synergistic interaction occurred when the two agents were used together.

It was interesting that BA was also confirmed to be highly related to cytotoxic activity against MDR cell lines (Jung et al., 2007; Saeed et al., 2018), whereas this does not necessarily mean that the processing was P-gp relevant. As reported, the inhibition of autocrine motility factor receptor (AMFR) activity should be the mechanism of BA to decrease MDR (Saeed et al., 2018). This could be a reasonable explication for the difference performance between BA and 23-HBA.

Indeed, previous report also suggested that 23-HBA could synergize the antitumor activity of ADR in multiple approaches (Zheng et al., 2010). However, the protein expression of P-gp was not modulated by 23-HBA during this process. Until now, the molecular mechanisms related to P-gp that underlie these processes remained unclear. To further explore the mechanism, intracellular accumulation was taken by measuring the transport of ADR and VCR in MCF-7/ADR or MCF-7/S cell lines (Figure 4). Similar to P-gp positive inhibitor verapamil, current study revealed that the intracellular accumulation of ADR and VCR, both P-gp substrates, were significantly promoted in MCF-7/ADR cells incubated with 23-HBA (0.2–20 μM) (Figures 4A,C); however, 23-HBA showed minor effect on the MCF-7/S cells (Figures 4B,D). These points are in line with the demonstrated effect of 23-HBA on MDR, suggesting that 23-HBA could change P-gp activity and transport to increase the intracellular accumulation of drugs.

In this study, the interaction mechanism between BA/23-HBA and P-gp was firstly investigated by multiple *in vitro* and in silico approaches. Both Caco-2 and MDCK-MDR1 cell models were firstly used to investigate the interaction between BA/23-HBA and P-gp. The active transport of VCR, known P-gp substrate, could be inhibited by 23-HBA instead of BA (Table 2). The results could reasonably explain why the MDR could be reversed by 23-HBA, but not BA (Figure 1). Furthermore, the weak inhibition of BA/23-HBA by VER in MDCK-MDR1 cells suggested both compounds are not a potent substrate of P-gp (Table 3), which was further confirmed by MDR1 shift approach (Figure 6). In molecular docking study could give the reason that the affinity of 23-HBA and BA with P-gp was different. The docking results indicated that amino acid residues in P-gp that interact with BA and 23-HBA were inconsistent (Figure 7). In addition, the combination mode and energy were also different. This further confirmed that the different pharmacokinetics and pharmacodynamics related with P-gp for the two compounds.

In conclusion, this *in vitro* study provides prospects that 23-HBA would greatly promote the efficiency of chemotherapeutic agent in P-gp resistant MDR cells. Furthermore, overcoming of MDR by 23-HBA was suggested to be related with P-gp inhibition. The *in vitro* and in silico study further revealed that 23-HBA and BA showed different interaction mechanisms with P-gp. Evidence of MDR reversal by 23-HBA would confirm the synergetic benefits of combining the product with other conventional anti-tumor agents in overcoming drug resistance in cancer chemotherapy.

## Data Availability

The original contributions presented in the study are included in the article/Supplementary Material, and further inquiries can be directed to the corresponding authors.
