# Supplementary material for: Involvement of P-gp on Reversing Multidrug Resistance Effects of 23-Hydroxybetulinic Acid on Chemotherapeutic Agents
Source: Front Pharmacol. 2021 Dec 15;12:796745. doi: 10.3389/fphar.2021.796745 (PMC8714961; doi:10.3389/fphar.2021.796745)
Supplement: Supplementary file 1 [file DataSheet1.DOCX]

**Supplementary 1**

2.10 Analysis method

For adriamycin, briefly, cell suspension (100 µl) was extracted by precipitation using 300 ml methanol followed by centrifugation, then the upper liquid (200 µl) was analyzed by Shimadzu LC10AD HPLC system (Shimadzu, Kyoto, Japan). The HPLC was set at 495/560 nm with a Kromosil ODS column (250×4.6 mm, 5 µm, Eka Chemicals AB, Sweden) under 40 °C. The mobile phase included 75% acetonitrile and 25% water (0.1% formic acid) with a flow rate of 1 ml/min.

For vincristine, briefly, cell suspension (200 µl) was extracted by addition of ethyl acetate (1 ml) and internal standard vinorelbine (10 µl). After 10 min centrifugation (3,000 g) , the organic supernatant (800 µl) was transferred into a plastic EP tube and dried at 37 °C. The residue was resuspended in acetonitrile (100ul). LC-MS was used to detecdted the concentration of the samples. The HPLC system was connected to a Shimadzu LC-MS-2010A quadrupole mass spectrometer with an electrospray ionization (ESI) in positive scan mode. Data acquisition were analyzed by Shimadzu LC-MS Solution software (Version 2.04). Chromatographic separation was performed on a Zorbax Extend C_18_ column (50mm×2.1 mm, 5 µm, Agilent, USA) with the mobile phase including of acetonitrile (A) and water with 0.1% formic acid (B). The linear gradient was from 25% A (v/v) for 0.5 min, to 92% A at 2.5 min, maintained at 92% A till 4 min, to 25% A at 4.3 min, and maintained at 25% A till 8 min. The selected ion monitoring (SIM) was used in the detection using [M+H]^+^ ions with best sensitivity for vincristine (m/z 825.3) and vinorelbine (m/z 779.35).

For BA and 23-HBA , briefly, sample (150 µl) was extracted by addition of ethyl acetate (1 ml) and internal standard digitoxin (10 µl). The other steps were taken according to the above process for vincristine. The mobile phase system included acetonitrile (A) and water with 0.0025% triethylamine and 0.0075% ammonium acetate (B). The linear gradient was from 40% to 60% A (v/v) at 0.5 min, maintained at 60% A till 1.8 min, to 90% A at 2.5 min, maintained 90% A till 6.5 min, and to 40% A at 9 min. The mass spectrometer was operated in negative ion and SIM mode with m/z 455.2 for BA, m/z 471.2 for 23-HBA, and m/z 763.5 for digotoxin were selected for quantification.

For digoxin, briefly, sample (150 µl) was extracted by addition of ethyl acetate (1 ml) and internal standard digitoxin (10 µl). The other steps were taken according to the above process. A Finnigan Surveyor™ HPLC system and Finnigan TSQ Quantum Discovery max system equipped with an electrospray ionization source (Thermo Electron, San Jose, CA) was used for analyzing. Data acquisition and processing were performed by Xcalibur 1.2. Chromatographic separation was carried out on a Luna-C_18_ column (150 mm×2.0 mm, 5 µm, Agilent, USA) with the mobile phase system consisting of methanol (A) and water with 0.1% ammonium acetate (B). The linear gradient was from 20% A (v/v) for 0.5 min, to 65% A at 0.8 min, maintained at 92% A till 3 min, to 20% A at 4.5 min, and maintained at 20% A till 8 min. The mass spectrometer was operated in negative ion and selective reaction monitor (SRM) mode with m/z 779.4-649.2 for digoxin and m/z 763.5-633.98 for digotoxin were selected to quantification.
